# Supplementary material for: Benefits of Respite Services on the Psycho-Emotional State of Families of Children Admitted to Hospice Palliative Care Unit: Preliminary Study on Parents’ Perceptions
Source: Healthcare (Basel). 2024 Mar 29;12(7):748. doi: 10.3390/healthcare12070748 (PMC11011478; doi:10.3390/healthcare12070748)
Supplement: Supplementary file 1 [file healthcare-12-00748-s001.zip › healthcare-2875655-supplementary.pdf]

Questionnaire on the perception of the use of the respite programme and its benefits

You are invited to participate in a survey that aims to assess your perceptions of using a respite care programme and the benefits it can bring to improving your psycho-emotional state.

As a result, we ask your permission to process the data resulting from the answers to the questionnaire you will fill in. All personal data will be kept confidential.

Current legislation on the ownership, use and protection of personal data - GDPR (Regulation 679/2016) will be respected for all study participants.

You are free to decide whether or not you would like to participate in this study.

I have read and understand this consent form and agree to voluntarily participate in the study described.

PART I

1. Place of residence:__________________________

2. Do you consider yourself to be a caregiver for a family member or friend?

☐ YES
☐ NO

Please read the definition of ***respite*** below and answer the questions below:

Respite is a short or long term break for an individual who is normally the permanent carer of a family member or friend in need. This concept is specifically designed to give the carer some time to themselves, away from the day-to-day responsibilities they normally have to the cared-for person. Respite can be offered both at home and away from home. It is usually offered by community organisations such as mental health centres, nursing homes, residential homes, churches or private providers. These organisations provide specialist paid staff or specialist volunteers to provide quality care for the sufferer and the respite needed by the carer.

3. Before reading this article, were you aware of the concept of respite?

☐ YES
☐ NO

4. Have you benefited from such respite services in the past?

☐ YES
☐ NO

5. What do you think are the main benefits of receiving respite services?
If you have never benefited from such services, please imagine what these benefits could be.
Please tick 3 benefits below:
☐Time to recharge, catch up on sleep or go on holiday;
☐Time to devote to my family or partner;
☐Time to attend sporting, artistic or cultural events;
☐Time to perform some of my duties;
☐Time to have my necessary medical check-ups or to accompany another family member to them;
☐Time for my hobbies or to pursue my studies;
☐It would help me to focus on my job and to keep it;
☐It would help prevent me from hurting myself or others due to overwork;
☐Time to learn or perfect my grooming techniques;
☐Time to seek guidance and assistance for stress and burnout, or to attend support groups;
☐Other:
____

6. In your opinion, how true is the following sentence? Tick **X** in one of the following columns:

If you have never benefited from respite services and do not have an answer, please tick the last column with ***NO KNOW***

	Very true	Partial true	Away from truth	False	I don't know
a. Respite services offer a safe and quality care					
b. Respite services reduce my psycho-emotional stress level					
c. Respite services help me to I improve my caring skills					
d. Respite services reduce the risk of neglect or inappropriate treatment of the family member or friend who needs my care;					
e. Respite services allow a friend or family member to receive care at home without having to move to a residential facility, which helps reduce costs. care;					
f.I do not trust the suppliers of respite services;					
g. I don't feel comfortable receiving strangers in the home;					
h. My family member or friend would not allow themselves to be cared for by another person besides me.					

7. What kind of respite care providers do you think exist in your area?
Please mark all that are available in your area:

- ☐ Home care
- ☐ Residential centre
- ☐ Day centres
- ☐ Hospice palliative care unit
- ☐ I don't know what kind of care exists in my area.

8. If you have ever been informed about respite services, where did you get this information?

- ☐ Friends, family, word of mouth;

- ☐ Recommendation from a doctor;
- ☐ Recommendations from the Disability Assessment Commission
- ☐ Recommendation on behalf of institutions providing social support
- ☐ Brochures/information leaflets
- ☐ Internet
- ☐ Other___________
- ☐ I don't remember
- ☐ I have never received such information

9. Have you ever had anyone help you find the contact details of such an institution?

- ☐ YES **(go to question 11a)**
- ☐ NO **(go directly to question 12)**
- ☐ NOT SURE (**go directly to question 12)**

10. Do you know how to find a respite provider that you can trust?

- ☐ YES
- ☐ NO

11a. If yes, who helped you?

- ☐ A friend or family member
- ☐ A healthcare provider
- ☐ Commission for the assessment of people with disabilities
- ☐ From an institution providing social services
- ☐ A religious organization
- ☐ Other:_________________

11. Do you know how you can tell if a respite service suits your needs?

- ☐ YES
- ☐ NO

12. Has it ever happened that you needed respite and didn't get it?

- ☐ Yes, I happened to need such a service, but I couldn't find one that suited my needs. **(please answer question 14a);**
- ☐ No, I have received this kind of service every time I needed it. **(go straight to question 15);**
- ☐ I have never tried such services. **(go straight to question 15);**

13. How often would you need such services?

- ☐ I don't need
- ☐ Less than once a month
- ☐ Twice a month
- ☐ More than 3 times a month

14a. If you've ever looked for such a service and not found one suitable, why do you think this happened? (choose 3 of the options below)

- ☐ I couldn't afford it financially;
- ☐ I was put on a waiting list;
- ☐ It was not available at the time I wanted;

- ☐ Services available do not address the age or special needs of the person they care for
- ☐ It was too far from my home

- ☐ Altele:____

14. How often have you received respite services in the last 6 months?

- ☐ I have not benefited from such services in the last 6 months
- ☐ Less than once a month
- ☐ Once or twice a month
- ☐ At least 3 times a month

15. When you need such services, is it easy or difficult to plan them?

- ☐ Very easy **(go straight to question 18)**
- ☐ Mostly easy **(skip to question 18)**
- ☐ Not sure **(go directly to question 18)**
- ☐ Mostly difficult **(please answer question 17a)**
- ☐ Very difficult **(please answer question 17a)**
- ☐ Not applicable-I have never benefited from such services **(go directly to question 18)**

16. If you were to benefit from such services when you need them, what effect would it have on the stress accumulated due to the responsibilities of being a primary caregiver?

- ☐ I'd be a lot less stressed
- ☐ Less stressed
- ☐ Equally stressed
- ☐ More stressed

17a. If you encountered difficulties in planning such services, why did this happen?
____
____
______

17. What were your coping strategies (actions, behaviours and thoughts you used to deal with the situation)?

- ☐ seeking help from friends
- ☐ positive thinking
- ☐ changing the way we see the situation
- ☐ seeking information, seeking another opinion, asking questions
- ☐ avoiding stressful situations, contact with the hospital or other family members who have not proven to be supportive.

Thank you for taking the time to answer the questions in the first part!

If you have a little more time, it would be helpful if you could answer a few more questions about yourself and your loved one in need of care. Your answers to these questions would help us to better understand the information you have already provided. However, if this seems too much, you can stop filling in this questionnaire now and return it to us.

PART II

Now we would like to ask you a few questions about the person you care for.

If you do not wish to answer certain questions, you do not have to.

18. How many people do you currently provide unpaid care to?

☐ 1
☐ 2
☐ 3 or more

If you provide such care to more than one person, please consider in the following the one that requires the most attention from you

19. How long have you been providing this care?

☐ Less than 1 year
☐ 1-3 years
☐ 4-10 years
☐ More than 10 years

20. What kind of help do you give this person?

☐ Company (discuss, read) or supervision
☐ Transport/escort for medical check-ups or where needed to reach staff
☐ Household help (cleaning, cooking, shopping)
☐ Personal care (feeding, washing, dressing)
☐ Medical care (performing treatment, wound care)
☐ Financial assistance (bill payments, budget planning)
☐ None of the above are specific :

____

21. How much time do you spend in a week to provide your care?

☐ 5 hours per week or less
☐ 6-20 hours per week
☐ 21-40 hours per week
☐ More than 40 hours per week

If you have received respite services in the past, please answer questions 23 and 24. If you have never benefited from such services, skip to question 25.

22. If you received respite services, who provided them?

- ☐ A relative or close friend
- ☐ A volunteer
- ☐ A home care provider
- ☐ Private medical care
- ☐ Mental Health Centre
- ☐ Emergency service
- ☐ Places of worship (Church)
- ☐ Day centre
- ☐ Camps for children
- ☐ Specialised centre for paediatric care
- ☐ Placement centre
- ☐ Other:___________

23. Where were these services provided to you?

- ☐ Home
- ☐ In a residential centre
- ☐ In a day centre
- ☐ Other:___________

24. What kind of relationship do you have with the person you care for?

- ☐ Husband/wife
- ☐ Parent
- ☐ Family relative
- ☐ Friend
- ☐ Child
- ☐ Other:___________

25. What is the main diagnosis of the person you care for?

- ☐ Chronic childhood encephalopathy. Spastic tetraparesis;
- ☐ Duchenne muscular dystrophy;
- ☐ Cancer or lymphoma;
- ☐ Congenital heart defect;
- ☐ Genetic disease;
- ☐ Hydrocephalus;
- ☐ Central nervous system and spinal cord malformations
- ☐ Plurimalformative syndrome;
- ☐ Other:___________
- ☐ I prefer not to answer

26. *Please provide us with the following information about yourself and the person you are caring for. If you do not wish to do so, please feel free not to fill it in.*

	**YOU**	**The person you care for**
**GENDER**	☐ FEMININE<br>☐ MASCULINE	☐FEMALE<br>☐MALE
**AGE**	☐ 19 YEARS OR YOUNGER<br>☐ 20-29 YEARS<br>☐ 30-39 YEARS<br>☐ 40-49 YEARS<br>☐ 50-59 YEARS<br>☐ 60-69 YEARS<br>☐ 70-79 YEARS<br>☐ OVER 80	☐Under 1 year<br>☐1 - 9 YEARS<br>☐10 -19 YEARS<br>☐20 -29 YEARS

Thank you for your time!
